# Nitrification and beyond: metabolic versatility of ammonia oxidising archaea

**DOI:** 10.1038/s41396-023-01467-0

**Published:** 2023-07-14

**Authors:** Chloe L. Wright, Laura E. Lehtovirta-Morley

**Affiliations:** School of Biological Sciences, University of East Anglia, Norwich, NR4 7TJ United Kingdom

**Keywords:** Environmental microbiology, Biogeochemistry, Metabolism, Archaeal physiology, Biogeochemistry

## Abstract

Ammonia oxidising archaea are among the most abundant living organisms on Earth and key microbial players in the global nitrogen cycle. They carry out oxidation of ammonia to nitrite, and their activity is relevant for both food security and climate change. Since their discovery nearly 20 years ago, major insights have been gained into their nitrogen and carbon metabolism, growth preferences and their mechanisms of adaptation to the environment, as well as their diversity, abundance and activity in the environment. Despite significant strides forward through the cultivation of novel organisms and omics-based approaches, there are still many knowledge gaps on their metabolism and the mechanisms which enable them to adapt to the environment. Ammonia oxidising microorganisms are typically considered metabolically streamlined and highly specialised. Here we review the physiology of ammonia oxidising archaea, with focus on aspects of metabolic versatility and regulation, and discuss these traits in the context of nitrifier ecology.

## Introduction

Our understanding of the global nitrogen cycle has been revolutionised in the last few decades thanks to the discovery of novel processes and microbial players. This includes the detection of anaerobic ammonia oxidising (anammox) bacteria in natural and engineered ecosystems [1–3] and, more recently, the characterisation of complete ammonia oxidation (comammox) by comammox *Nitrospira*, which oxidise both ammonia and nitrite within the same cell to produce nitrate [4, 5]. Perhaps the most unanticipated breakthrough though, was the discovery of aerobic ammonia oxidation within the domain Archaea nearly two decades ago [6, 7]. Ammonia oxidising archaea (AOA) constitute a major fraction of microbial biomass on Earth [8] and play a vital role in the global biogeochemical cycling of nitrogen. Understanding the drivers of ammonia oxidation in the environment is of major importance since it could contribute towards food security and help mitigate the release of the greenhouse gas nitrous oxide (N_2_O) and nitrate (NO_3_^-^), which play roles in climate change and groundwater pollution, respectively [9]. Whilst significant progress has been made into cultivation and physiology of AOA, as well as environmental factors affecting their distribution and activity, many knowledge gaps still remain in our understanding of their metabolism, cellular regulation and adaptation to environmental changes. Ammonia oxidisers, including AOA, are autotrophic and able to fix their own carbon from inorganic CO_2_ and to generate reductant from ammonia [6, 10]. They are considered specialists in this specific metabolism. Here we review the physiology and metabolism of ammonia oxidising archaea, including whether AOA are as metabolically constrained as often thought. This is an ever-unfolding field of research as more ammonia oxidisers are isolated or highly enriched in culture, and novel insights into their metabolism revealed.

## Ammonia oxidation kinetics: the roles of ammonia and oxygen

Ammonia (NH_3_), rather than ammonium (NH_4_^+^), is the substrate oxidised by the key enzyme ammonia monooxygenase (AMO) from the AOB *Nitrosomonas europaea* [11]. The current consensus is that ammonia is also the preferred substrate for the archaeal AMO [12]. Ammonia oxidisers are widely considered to be specialist microorganisms, for whom ammonia is usually the sole source of reducing power [4–6, 13]. It is therefore not surprising that the affinity for ammonia has been a subject of intensive research [12, 14, 15]. Ammonia concentration is a key eco-physiological factor influencing the abundance and distribution of nitrifiers. Early kinetics studies suggested that AOA and comammox bacteria are adapted to low ammonia concentrations, although it is now known that ammonia oxidisers exhibit a wide range of ammonia affinities. Within AOA, the *K*_m(app)_ for ammonia ranges across four orders of magnitude from representatives of genus *Nitrosocosmicus* with the highest K_m_ values (>12 µM), comparable to many characterised soil AOB [16–19], to the lowest values in *Nitrosopumilales* and ‘*Ca*. Nitrosotaleales’ genera with *K*_m(app)_ in the low nM range (<2.8 nM) [12, 14]. In addition, there could be differences in the kinetics between AOA strains, but potentially also depending on growth conditions. For instance, *Nitrosocosmicus*-affiliated AOA outcompeted other ammonia oxidisers in ammonia-limited soil enrichments, suggesting that some members of this genus are adapted to low ammonia concentrations at least under certain conditions [19]. Although cultured representatives of genus *Nitrosocosmicus* have a high tolerance to ammonia compared to other AOA [16–19], there is no indication that high ammonia tolerance is necessarily linked to low ammonia affinity.

Ammonia and ammonium exist in pH-dependent equilibrium with a pK_a_ of 9.25, meaning that ammonia decreases exponentially with decreasing pH. Laboratory cultures usually have ample supply of ammonia, but nitrifiers in the environment are frequently exposed to low and fluctuating substrate concentrations [6, 20]. Affinity for ammonia is thus likely to be an important factor for survival in the environment [6, 20]. A high affinity for ammonia would be advantageous to nitrifiers found in environments with low ammonia concentrations or low pH. The ammonia oxidation kinetics are also relevant to agriculture and engineered systems [21–25]. Nitrifiers, denitrifiers and anammox bacteria often integrate themselves into biofilms, where diffusion causes gradients in ammonia concentrations [11, 26, 27]. This means that microorganisms embedded deeper in the biofilm may experience lower ammonia concentrations. Subsequently, ammonia availability can influence competition and cooperation between these communities [26–29].

Whilst more research efforts have focused on the affinity for ammonia, the AMO has two substrates: ammonia and oxygen. Oxygen plays an important role in nitrification both as a substrate for the AMO enzyme and as a terminal electron acceptor for ammonia oxidisers [26]. Even though oxygen is a substrate for AMO, it is often overlooked, despite the potential value of characterising kinetic parameters and linking to niche, as has been done with ammonia. In marine environments, AOA of the family *Nitrosopumilaceae*, particularly those associated with the marine low ammonia ecotype, are successful under oxygen-limited conditions, with the highest abundances often detected in oxygen minimum zones (OMZs) and deep ocean sediments [30, 31]. Rates of ammonia oxidation in OMZs were measurable at <0.01 μM O_2_, likely driven exclusively by communities of AOA [31]. The enrichment of AOA from marine sediments was attributed to their ability to outcompete co-occurring bacteria at low O_2_ [32]. Recently, it was demonstrated *Nitrosopumilus maritimus* SCM1 was able to generate small amounts of oxygen under anoxic conditions, most likely by nitric oxide disproportionation [33]. Although the potential oxygen production by other AOA strains or in the environment is not yet fully explored, the ability to produce oxygen may provide an explanation for the presence of AOA in oxygen-limited habitats such as OMZs. In *N. maritimus*, some of the oxygen produced is used by the aerobic metabolism, which includes both the ammonia oxidation pathway and the respiratory chain, and some oxygen is released from the cells [33]. The metabolic pathway of oxygen production in *Nitrosopumilus maritimus* is not yet fully resolved, but involves nitric oxide and nitrous oxide [33, 34]. Many AOA encode chlorite dismutase-like enzymes [35], however, the function of these in AOA is unknown. Chlorite dismutase from the NOB “*Candidatus* Nitrospira defluvii” has been shown to reduce chlorite to chloride and O_2_ [36, 37]. Further studies are required to test the impact of oxygen producing metabolism in the environment. Affinity for oxygen and the ability to produce oxygen could influence niche specialisation and competition between different groups of ammonia oxidisers. For instance, comammox bacteria are well-adapted to the low oxygen concentrations in the oxic-anoxic interface of the biofilms and have been enriched from bioreactors under low dissolved oxygen conditions [3, 4, 38, 39]. AOA from wet tropical soils were resistant to prolonged intervals of anoxia [40] and reacted faster to anoxic/oxic fluctuations compared to AOB [41]. AOA seem to have a somewhat higher affinity for oxygen than AOB, with *Nitrosopumilus* representatives having the highest affinity (Table 1). However, studying the affinity for oxygen in further AOA, AOB and comammox strains and mixed communities, could provide insights into how oxygen availability shapes nitrifying communities.

**Table 1:** Oxygen uptake kinetics by ammonia oxidisers.

Strain	*K*_m_ (μM)	*V*_max_ (μmol O_2_ mg prot^−1^ h^−1^)	Reference
AOA			
*Nitrosarchaeum koreense* MY1	10.4 (1.1)	20	[37]
*Nitrosopumilus maritimus* SCM1	3.9 (0.6)	36	[15]
*Nitrosopumilus* AR Enrichment	2.0 (0.5)	11	[30]
Ca. Nitrososphaera sp. JG1	4.7 (0.2)	35	[38]
Ca. Nitrosocosmicus franklandus C13	11.5 (1.9)	N.D.	Lehtovirta-Morley, unpublished
AOB			
*Nitrosomonas europaea* (ATCC 19178)	1.3–14.9	N.D.	[39]
*Nitrosomonas europaea* C-31 (ATCC 25978)	186	129	[30]
*Nitrosomonas mobilis* Ms1	21.7 (4.0)	N.D.	[40]
Activated sludge	15.6	N.D.	[41]

*N.D.* not determined.

Despite intense research on ammonia oxidation kinetics over the past few years, there are still outstanding questions, particularly with regards to mechanisms which underpin the whole-cell kinetics. Structural basis of AMO conferring high or low affinity is not understood, nor is the role of ammonia transport. Furthermore, although AMO is known to be a membrane-bound enzyme [13, 42], the location and orientation of the AMO active site remain open research questions. In addition, the role of oxygen in AOA is understudied and could shed light on the niche adaptation of ammonia oxidisers in the future.

## Nitrogen uptake and metabolism

### Ammonium transport mechanisms

**Fig. 1:**
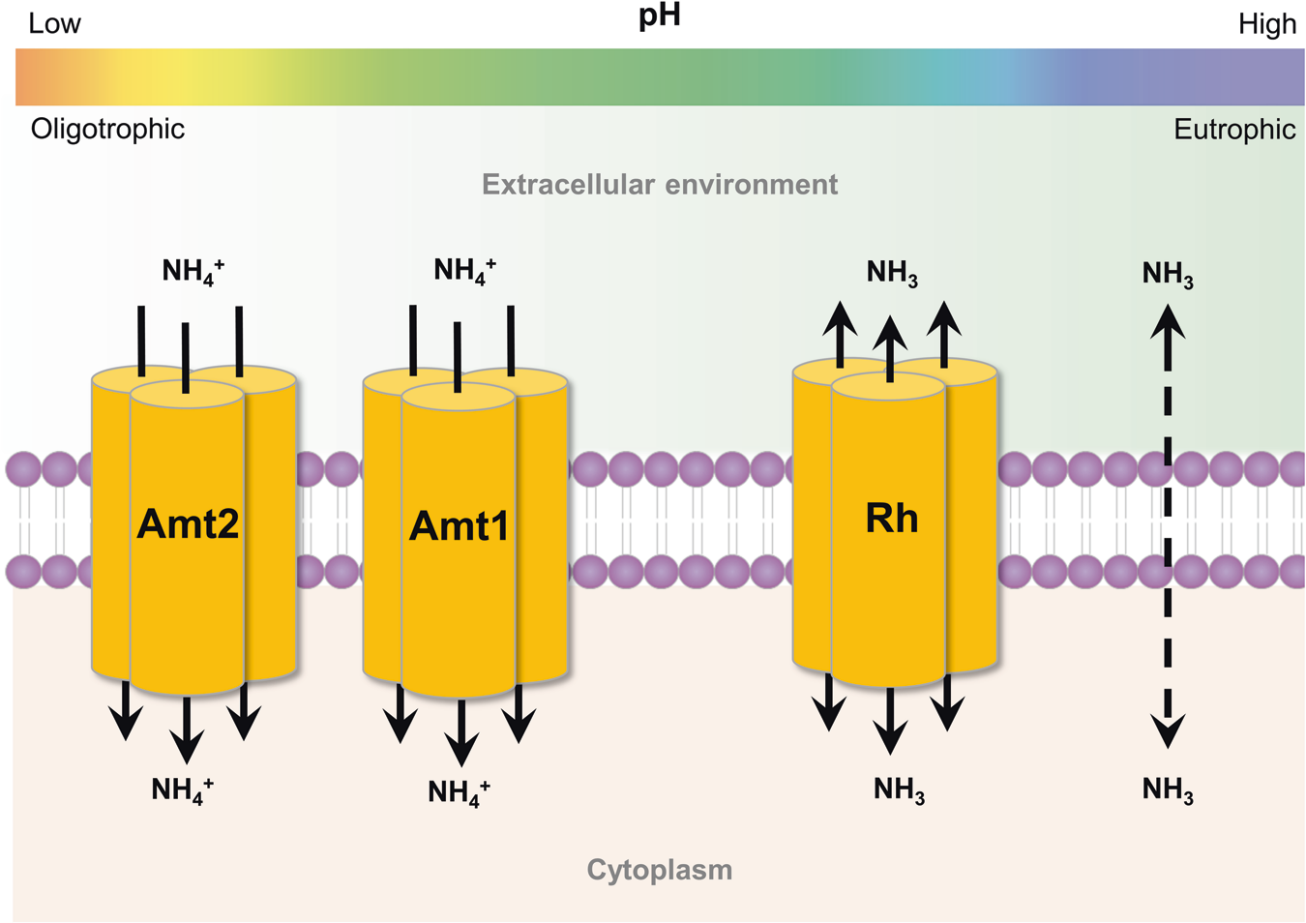
Amt and Rh-type transporters encoded by ammonia oxidising microorganisms. Amt transporters are energy-dependent and bind ammonium. Amt2 is a high-affinity Amt and can function at low pH and substrate concentration. Rh proteins facilitate the bidirectional flow of NH_3_ and therefore function at high pH and substrate concentration. NH_3_ can also cross the bilipid membrane by diffusion. NH_4_^+^ and NH_3_ exist in equilibrium based on pH. Membrane permeability between AOA and AOB may differ as their membrane compositions are different [60].

Much research focus has been on ammonia oxidation, but ammonium is required for both energy metabolism and assimilation in ammonia oxidisers. There must presumably exist reasonably sophisticated regulation for ammonium uptake, assimilation, and oxidation, particularly because ammonia is a relatively poor energy-yielding substrate. An overwhelming majority of ammonia is used for energy metabolism rather than anabolism, indicated by the near stoichiometry of 1:1 for ammonia:nitrite typically observed for ammonia oxidation [6, 13]. It is not known, whether ammonium transport is in any way coupled to oxidation in AOA, or if the transport is solely required for assimilation. Ammonia can cross biological membranes, but many organisms rely on the import of ammonium to meet nitrogen demands. Ammonium transport is mediated by a class of ubiquitous membrane proteins comprising ammonium transporters (Amts), methylammonium permeases (Meps) and rhesus (Rh) proteins [43, 44]. Many previously characterised Amts function as energy-dependent electrogenic ammonium transporters whilst the Rh-type proteins act as low-affinity ammonia channels [43, 45]. Mechanism of ammonium transport has been studied for decades and is still not fully understood. Recently, a two-lane mechanism was demonstrated for electrogenic ammonium transport by *Escherichia coli* AmtB and *Nitrosomonas europaea* [46]. Active transport against an ammonium gradient would presumably require energy, although energetics of ammonium transport remain enigmatic and intracellular ammonium concentrations in AOA are not known. Ammonia oxidation provides low energy yield, and active transport would be energetically costly [13]. On the other hand, active transport of ammonium could be advantageous to nitrifiers in acidic habitats and nitrogen-limited environments (Fig. 1), because, despite requiring energy, it could enable these nitrifiers to colonise otherwise inaccessible niches.

Most AOA encode at least two Amt-type transporters, whilst approximately half of the available AOB genomes contain Rh proteins [47]. Other AOB lack recognisable transporters and presumably rely on ammonia diffusion [47]. The two different clades of comammox bacteria appear to utilise distinct ammonium uptake mechanisms. Clade A encode Rh-type transporters with >70% amino acid similarity to those of the β-AOB, whereas clade B encode Amt-type transporters [48]. Anammox bacteria encode both types of transporters [49] (Supplementary Fig. S1). The Rh protein (Rh50) from *Nitrosomonas europaea* and Amt5 from ‘*Ca*. Kuenenia stuttgartiensis’ have both been isolated by recombinant expression and structurally characterised [50, 51]. Rh50 from *Nitrosomonas europaea* has been experimentally demonstrated to function as an ammonia transporter [46, 50, 52]. Electrophysiological analysis of ‘*Ca*. Kuenenia stuttgartiensis’ Amt5 revealed no transport function and instead this protein acts as an ammonium sensor [51]. Sequence and structural dissimilarities between the ammonium transporters in AOA and bacterial ammonia oxidisers indicate that they are functionally distinct [53]. Additionally, the transcriptional response of the archaeal Amt transporters to different ammonia concentration suggests they operate as high- and low-affinity transporters [54–56]. All sequenced representatives of the *Nitrosocosmicus* genus only encode one low-affinity Amt [16–18, 57]. Additionally, these strains lack the S*-*layers, which can function as ammonium concentrating mechanisms [58, 59]. The affinities of archaeal ammonium transporters have been inferred from transcriptomic studies, but not yet tested directly [56]. In addition, the role of the thaumarchaeal cell envelope in concentrating ions remains underexplored, as does the regulation of how ammonia is partitioned for assimilation and oxidation by AOA. Furthermore, archaeal and bacterial membranes have distinctly different compositions, which may affect membrane permeability and the rate at which ammonia can diffuse [60], although this question has not yet been fully explored.

### Ammonia assimilation pathways

**Fig. 2:**
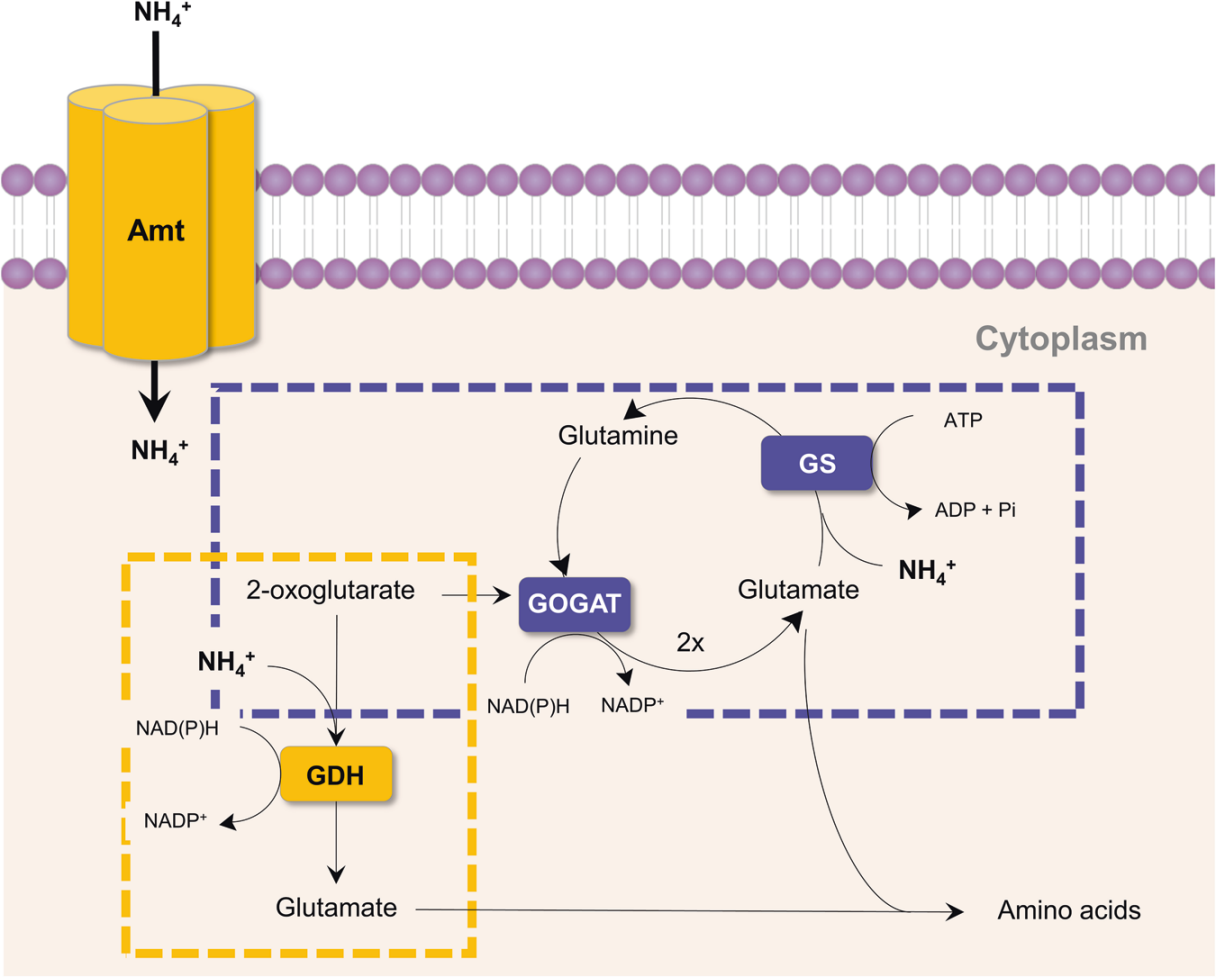
Ammonia assimilation pathways. The glutamate dehydrogenase (GDH) pathway, represented in yellow, has a low-affinity for NH_4_^+^ and is found in all sequenced AOA. The high-affinity glutamine synthetase-glutamate synthase (GS-GOGAT) pathway is represented in purple and is only present in some representatives of the *Nitrosocosmicus* genera. One ATP is required per NH_4_^+^.

All characterised AOA contain glutamate dehydrogenase (GDH) and glutamine synthetase (GS) [57]. GDH is a key enzyme in ammonia assimilation and catalyses the reversible reductive amination of 2-oxoglutarate to glutamate [57, 61] (Fig. 2). In heterotrophs, this low-affinity pathway is favourable under energy and carbon limiting conditions since no ATP is consumed and less carbon is used per ammonia molecule assimilated [62]. The role of GDH in AOA is understudied. It seems likely that intracellular ammonium concentration and the regulation between ammonia uptake, assimilation and oxidation would be important for the function of this pathway in AOA, although this currently remains untested. GS catalyses an ATP-dependent conversion of ammonia and glutamate into glutamine and is considered to play an important role in central nitrogen metabolism. Nearly all AOA contain P_II_ signal transduction protein homologues which belong to the glnK/B subfamily and regulate nitrogen metabolism [60, 63]. GlnK and GlnB interact directly with Amt transporters and glutamine synthetase (GS), respectively, to regulate ammonium influx into the cell by uridylylation of P_II_ in response to low ammonia concentration, and also GS activity in response to extracellular and intracellular nitrogen concentrations [63]. The external ammonia concentration is likely important for ammonia assimilation, and the transcriptional activity of both GS and GDH in *Nitrosocosmicus agrestis* was upregulated in response to high ammonia concentrations [64].

Two known AOA, *Nitrosocosmicus arcticus* and *Nitrosocosmicus oleophilus*, encode genes for the glutamine synthetase-glutamate synthase (GS-GOGAT) pathway [17, 57]. In this pathway, GS which is conserved in AOA, converts glutamate to glutamine. Glutamine-oxoglutarate amidotransferase (GOGAT) catalyses the NADPH-dependent formation of two glutamate molecules from glutamine and 2-oxoglutarate (Fig. 2). This high affinity, energy consuming pathway for ammonia assimilation functions well at low ammonia concentrations, and when the cell is not energy or carbon limited [61]. The reason for the presence of GS/GOGAT in some AOA is unknown as are many details of ammonia assimilation in AOA. It is possible that most AOA use GDH, as this pathway is energetically less costly than GS/GOGAT, even if it requires high concentrations of ammonia. It was also postulated that some *Nitrosocosmicus* strains might have as-yet unidentified, auxiliary energy sources, which may explain their use of ATP-dependent GS/GOGAT pathway [57].

## The biochemistry of energy metabolism of AOA

### The structure, function, and substrate range of the AMO

**Fig. 3:**
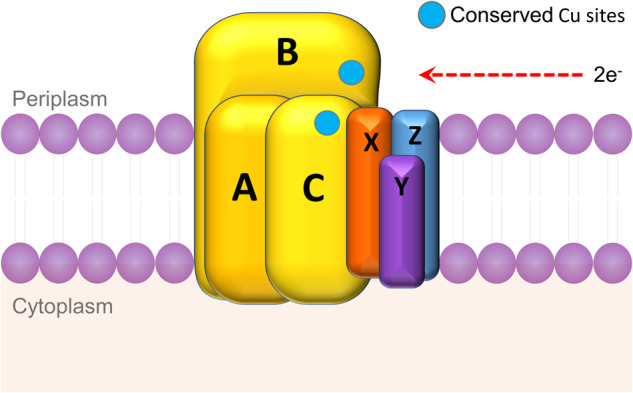
Schematic of the archaeal ammonia monooxygenase (AMO). AmoA, AmoB, and AmoC (yellow), are conserved and form trimers (AmoABC). AmoB and AmoC contain copper binding sites, which are conserved in AOA and AOB. AmoX (orange), AmoY (purple), and AmoZ (blue), are putative archaeal AMO accessory proteins.

The AMO is a copper-dependent multimeric transmembrane enzyme belonging to the CuMMO superfamily which comprises of ammonia, methane, and alkane monooxygenases [4–6, 13, 42]. Based on the similarities to bacterial AMO and pMMO, it is assumed that the oxidation of ammonia by the archaeal AMO requires two electrons [13]. These electrons are provided via electron carriers from the downstream pathway, the oxidation of hydroxylamine, and possibly nitric oxide, although the role of nitric oxide in AOA is not fully understood [65, 66]. Assuming that the archaeal AMO functions similarly to its bacterial counterparts, two net electrons per ammonia molecule are generated from the ammonia oxidation pathway and this reductant powers the ATP synthesis and cellular anabolism, including carbon fixation [13]. Nitrous oxide is also produced by AOA during ammonia oxidation, and in ^15^N-labelling studies with *Nitrososphaera viennensis*, nitrous oxide was generated through N-nitrosating hybrid formation [67]. A recent on study on *Nitrosopumilus maritimus* found that, although hybrid formation is a key mechanism of nitrous oxide production, there are multiple pathways through which the constituent atoms of nitrous oxide are derived from ammonia, nitrite, O_2_ and H_2_O [68]. In addition, *Nitrosopumilus maritimus* can produce nitrous oxide through NO dismutation under anoxic conditions [31]. Active AMO is difficult to purify and many predictions about the structure have been based on homology to the better-characterised particulate methane monooxygenase (pMMO) from methanotrophs [69, 70]. The AMO is predicted to exist as a heterotrimeric complex composed of three subunits in bacteria: AmoA, AmoB, and AmoC [71]. The archaeal AMO is very divergent from bacterial AMO and other CuMMOs and appears to have additional subunits including AmoX, AmoY, and AmoZ [42] (Fig. 3). The location and nature of the AMO active site has not been identified. Analysis of the AmoB and AmoC protein structure favours an extracellular active site (outwards facing) [47], which would be logical considering the toxicity of hydroxylamine. The location and nature of the pMMO active site also remain uncertain, although mono- and di-copper sites have been proposed to reside in the soluble region of the PmoB [72] and a newly discovered tri-copper site is found in the PmoC subunit, close to the periplasm [73]. Mutagenesis studies on the hydrocarbon monooxygenase, a member of the CuMMO superfamily, in *Mycobacterium* NBB4 have demonstrated that the metal-binding residues on the C subunit are essential for activity [74]. Substrates and inhibitors of the AMO are largely non-polar, suggesting the active site is hydrophobic, and consistent with ammonia rather than ammonium as the natural substrate [75].

Copper is thought to be a co-factor in both the archaeal and bacterial AMO. The respiratory chain in AOA is predicted to be copper-based, and they contain numerous small blue copper proteins and multicopper oxidases which may be involved in electron transfer [61, 76]. In contrast, the respiratory chain and key enzymes e.g. hydroxylamine dehydrogenase (HAO) in AOB use proteins which require heme [13]. Nevertheless, it is estimated that both iron and copper may be limiting factors to the growth of AOA in the ocean [77, 78]. There are no known examples of AOA producing chalkophores (copper-binding molecules), and *Nitrososphaera viennensis* has a higher affinity for copper uptake than *Nitrosopumilus maritimus* does [78, 79]. Translating culture-based findings to the environmental context is challenging because copper bioavailability in many habitats is affected by factors such as pH and complexation with organic molecules [80].

Due to the difficulty in purifying AMO in its active state, much of what is known about the AMO has been discovered using inhibitors. Acetylene is a well characterised inhibitor of both the AMO and pMMO [81, 82]. With *Nitrosomonas europaea*, acetylene acts as a suicide substrate and cells require de novo protein synthesis of new AMO to re-establish ammonia-oxidising activity [83]. Incubations with ^14^[C]-acetylene resulted in the covalent radiolabelling of *Nitrosomonas europaea* AMO, enabling identification of the genes coding for AMO [84]. A subsequent study found that the ketene product of acetylene activation bound covalently to a histidine residue (H191) on the AmoA subunit of *Nitrosomonas europaea*, a residue thought to be in close proximity to the putative active site [85]. While acetylene is also an irreversible inhibitor of the archaeal AMO [86], the AMO from archaea lack the histidine residue responsible for binding in *Nitrosomonas europaea*, suggesting that acetylene must bind at a different position on the enzyme [85].

Insights into the structure of the archaeal AMO active site(s) and its potential substrate range has been provided by characterising the inhibition of archaeal AMOs to linear 1-alkynes [87–89]. The archaeal AMO demonstrates a reduced sensitivity to inhibition by larger 1-alkynes compared to bacterial AMO, suggesting they have a narrower hydrocarbon substrate range [87–89]. In fact, archaeal 1-alkyne inhibition profiles were similar to that of pMMO which can only oxidise linear C_1_-C_5_ alkanes and alkenes [88, 89]. Recent reconstitution of the pMMO in a lipid bilayer revealed the PmoC tri-copper binding site, which is adjacent to a hydrophobic cavity capable of accommodating up to C_5_ linear hydrocarbons [73, 90]. The aromatic alkyne, phenylacetylene, inhibited the archaeal and bacterial AMO at different threshold concentrations and by different mechanisms of inhibition, highlighting functional differences between the archaeal and bacterial AMO [89]. Kinetic analysis of the inhibition of ammonia oxidation by *Nitrosomonas europaea* demonstrated that unlike acetylene, phenylacetylene does not compete with ammonia for the same binding site and behaved as an uncompetitive inhibitor, suggesting phenylacetylene only had affinity for the AMO-ammonia complex [89]. Phenylacetylene inhibition of ‘*Ca*. Nitrosocosmicus franklandus’ was found to be non-competitive [89]. The results indicate the presence of secondary, non-ammonia, binding sites on both the archaeal and bacterial AMO, as previously suggested for the AMO from *Nitrosomonas europaea* and the pMMO [91–93].

It is proposed that the downstream metabolism refines the functional role of microorganisms containing CuMMO [94]. For example, the bacterial ammonia oxidisers *Nitrosococcus oceani* and *Nitrosomonas europaea* can oxidise methane but lack necessary downstream enzymes to gain energy from methane oxidation [95]. Likewise, several methanotrophs have been shown to co-oxidise ammonia, but this does not support growth [96]. AMO- and pMMO-expressing microorganisms have received interest for their potential use in bioremediation due to their capability to co-oxidize persistent organic pollutants such as halogenated alkanes and alkenes and chlorinated hydrocarbons [97, 98]. It was shown that ‘*Ca*. Nitrososphaera gargensis Ga9.2*’* was capable of co-metabolising two tertiary amines, mianserin and ranitidine (pharmaceutical drugs), with the initial oxidative reaction possibly carried out by AMO [99]. Co-oxidation of compounds other than ammonia by AOA is an open question and has not been fully explored yet. The work on alkyne inhibitors suggests that the substrate range for archaeal AMO may include hydrocarbons with chain-lengths <C_5_. If AOA participate in co-oxidation of such compounds e.g. methane, ethane, or propane, or even more complex branched hydrocarbons, this would be of importance for both bioremediation and for understanding global biogeochemical carbon cycling.

## Metabolic versatility

**Fig. 4:**
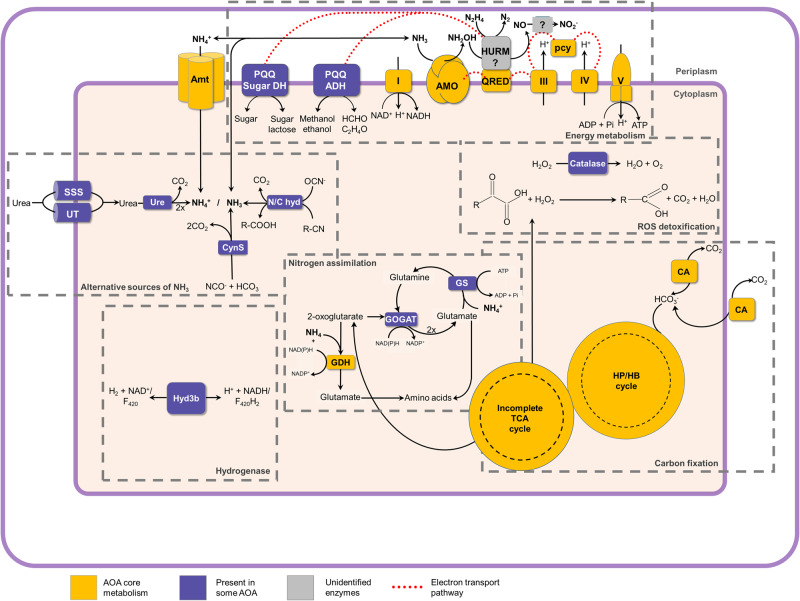
Schematic of predicted metabolic features from the genomes of AOA. Yellow represents genomic features encoded by all AOA. Blue indicates metabolisms not shared by all AOA. AMO ammonia monooxygenase, QRED quinone reductase, HURM hydroxylamine ubiquinone redox module, PQQ pyrroloquinoline quinone, DH dehydrogenase, ADH alcohol dehydrogenase, Amt ammonium transporter, UT urea transporter, SSS solute/sodium symporter, Ure urease, N/C hyd nitrile/cyanide hydratases, Cyn cyanase, TCA tricarboxylic acid cycle, HP/HB hydroxypropionate/hydroxybutyrate cycle, CA carbonic anhydrase, Hyd hydrogenase, GDH glutamate dehydrogenase, GS-GOGAT glutamine synthetase-glutamate synthase, ROS reactive oxygen species.

Autotrophic ammonia oxidising microorganisms are generally considered metabolically streamlined, and specialised in using ammonia as their sole source of energy. In contrast, several nitrifying microorganisms demonstrate remarkable metabolic flexibility [100]. Best known examples are nitrite-oxidising bacteria, which can derive energy for growth from formate, hydrogen and sulfide [100]. Some, but not all, ammonia oxidisers can use urea or cyanate as the sole source of energy and reductant as both are enzymatically converted to ammonium [101–104] (Fig. 4). Growth on urea or cyanate is not pH-dependent and therefore may be advantageous in acidic and low ammonium environments [105, 106]. Urea uptake is mediated by specific urea transporters (UTs) and solute/sodium symporters (SSS (DUR3)), after which urea hydrolysed intracellularly by a urease [107]. SSS transporters are phylogenetically divided into several distinct clusters, of which AOA share the closest sequence similarity with the plant urea transporters [107]. Urea uptake systems and urease enzymes have been reported in AOB, AOA and comammox bacteria [61, 101–103]. The hydrolysis of urea can support the growth of AOB such as *Nitrosoglobus terrae* and *Nitrosospira* sp. at low pH [105, 108]. However, the use of urea by marine AOA is not directly related to pH unlike often reported for ammonia oxidisers in acidic soils [102]. Urea is commonly present in marine habitats, and being able to use urea in addition to ammonia may give these AOA a competitive advantage [109]. *Nitrososphaera gargensis* Ga9.2 is currently the only genome-sequenced AOA that encodes a known cyanase, which catalyses conversion of cyanate to ammonium and CO_2_ [104]. *Nitrosopumilus maritimus* lacks a canonical cyanase, but also produces ammonia from cyanate, which suggests there must exist an as-yet unknown mechanism, which breaks down cyanate in *Nitrosopumilus maritimus* [109]. Genes putatively encoding for enzymes of a novel class of nitrilases or cyanide hydratases are found in genomes of AOA from the *Nitrosocaldus, Nitrosotenuis* and *Nitrosopumilus* genera [110–112]. Nitrilases catalyse the conversion of nitriles to the corresponding acid and cyanide hydratases convert hydrogen cyanide (HCN) to formamide, both of which can produce ammonia [112]. These homologues might be involved in the conversion of cyanate to ammonia, but their function has not yet been experimentally proven (Fig. 4).

Hydroxylamine is a physiological intermediate in the archaeal ammonia oxidation pathway [113]. No HAO homologue exists in AOA, nor do they have the genetic repertoire to fully synthesise *c-*type hemes. Therefore, AOA have a novel enzymology for the oxidation of hydroxylamine [113]. Hydrazine, a key intermediate in anammox catabolism and a structural analogue of hydroxylamine [114], was found to be an inhibitor of hydroxylamine oxidation by AOA [115]. In addition, the AOA isolate “*Ca*. Nitrosocosmicus franklandus” oxidised hydrazine to dinitrogen, with O_2_ consumption coupled with ATP production [115] (Fig. 4). Hydrazine is also a substrate for the bacterial HAO, and is oxidised to N_2_ (Eq. (1), [116]).

1 $$N_2H_4 \to N_2 + 4H^ + + 4e^ -$$

Electrons derived from this reaction can serve as reducing equivalents for the AMO and hydrazine has been used as an external source of reductant to fuel alternative substrate oxidations by *Nitrosomonas europaea* [117–119]. Ammonia, urea, cyanate, hydroxylamine, and hydrazine are currently the only experimentally confirmed energy-yielding substrates in AOA. However, there are many other predicted, as-yet unproven types of metabolism in AOA as discussed below.

Ammonia oxidisers are chemolithoautotrophs and able to fix their own carbon. Ammonia oxidising archaea fix their carbon using the 3-hydroxypropionate/4-hydroxybutyrate pathway and reductant generated from ammonia oxidation [10]. Nevertheless, there are some indirect indications of mixotrophic growth in ammonia oxidisers. Early studies of carbon metabolism in marine archaea indicated both autotrophic and heterotrophic modes of carbon assimilation occur, although it is possible that not all archaea in these environmental samples were AOA [120, 121]. A recent study found that in the Pacific Ocean, a proportion of the Marine Group I archaea, which includes AOA, incorporated auxiliary carbon from urea and amino acids [122]. However, it can be challenging to disentangle mixotrophy and autotrophy, because e.g. urea could be metabolised into CO_2_ and fixed autotrophically, resulting in indirect incorporation. In addition, a discrepancy between a high abundance of AOA marine water column and a low nitrification rate was reported [30]. One possible explanation for this observation would be mixotrophic growth, where AOA would use electron donors other than ammonia or fix carbon from alternative organic carbon sources instead of CO_2_. However, the only direct evidence of mixotrophy in ammonia oxidisers is known in *Nitrosomonas europaea*, the growth of which is stimulated by fructose and pyruvate which are assimilated as carbon sources [123]. There have been efforts to investigate the potential of AOA for mixotrophy using organic acids, some of which are tricarboxylic acid cycle intermediates [18, 124, 125]. However, the apparent stimulation of catalase-negative AOA by α-keto acids is due to the alleviation of oxidative stress, rather than mixotrophic growth [126] (Fig. 4). In addition, organic compounds can inhibit some AOA strains, potentially due to their high copper complexation potential or their toxic effect at low pH [80, 125].

The genome of “*Ca*. Nitrosocosmicus hydrocola” encodes for genes putatively involved in one-carbon (C_1_) metabolism, including methanol oxidation to formaldehyde and formate oxidation to CO_2_ [18]. Some AOA from deep seafloor sediments have the genetic repertoire to perform proteolysis and deamination to regenerate ammonia, coupling mixotrophic and autotrophic metabolisms [127]. Intriguingly, the growth of the recently described AOA “*Ca*. Nitrosocosmicus arcticus” was uncoupled from ammonia oxidation, suggesting that this strain has alternative or supplementing energy metabolism(s) [57]. The genomes of some “*Ca*. Nitrosocosmicus*”* strains encode putative periplasmic or membrane-bound pyrroloquinoline quinone (PQQ)-dependent dehydrogenases, which oxidise sugars/alcohols by simultaneously reducing electron acceptors, potentially contributing reducing equivalents to the respiratory chain [57] (Fig. 4). PQQ-dependent dehydrogenases were among the most highly expressed genes by the newly discovered heterotrophic marine thaumarchaea, and therefore are likely to be important for energy metabolism since these *Thaumarchaeota* lack the ability to oxidise ammonia [128].

Some AOA genomes contain coding sequences related to hydrogenases, although their function has not been verified in any AOA. The genomes of the thermophilic AOA “*Ca*. Nitrosocaldus cavascurensis” and “*Ca*. Nitrosocaldus islandicus” both contain genes encoding for the four subunits of a putative cytoplasmic Group 3b [NiFe]-hydrogenase [35, 129] (Fig. 4). Some previously characterised members of Group 3b hydrogenases are oxygen-tolerant and bidirectional, and can couple oxidation of H_2_ to reduction of NAD(P), or oxidation of NAD(P)H to fermentative production of H_2_ [130–132]. In addition, Abby and colleagues also speculate that oxidised F_420_ could be a potential cofactor for the 3b-[NiFe]-hydrogenase in “*Ca*. Nitrosocaldus cavascurensis”, although this has not yet been tested experimentally [129]. All members of genus ‘*Ca*. Nitrosocaldus’ are thermophilic and thrive in hot springs at temperatures of ~70 °C, although the link between the growth temperature and presence of Group 3b [NiFe] hydrogenases in AOA remains unproven. Hydrogenases appear to be mainly absent in the genomes of AOB, apart from two representatives from the *Nitrosomonas* cluster 6a and *Nitrosospira multiformis* [103, 133], both of which originate from soil ecosystems and contain putative Group 3d hydrogenases. Group 3b hydrogenases are also found in comammox *Nitrospira*, predominantly in the representatives from clade A [101]. The genomes of other AOA (including some representatives of genera *Nitrosotalea*, *Nitrososphaera* and *Nitrosocosmicus*) contain genes with homology to Group 4a [NiFe]-hydrogenases. These proteins, termed energy conserving hydrogenase-related complexes (Ehr), lack the CxxC motif required for hydrogenase activity and might play another role in electron transfer [134]. Ehr complexes are also found in comammox *Nitrospira* [135, 136]. It is unknown whether the Ehr complexes confer any advantages in terms of metabolic flexibility and environmental adaptation in ammonia oxidisers.

## Outlook

This review aimed to highlight some key knowledge gaps in AOA research, aside from the seemingly elusive enzymology of ammonia oxidation pathway and its intermediates [137, 138]. Ammonia oxidisers are widely regarded as relatively inflexible, but we have covered some possibilities of alternative metabolisms, including predicted pathways and potential co-oxidation, which require further exploration. In contrast to the laboratory, where culture conditions can be carefully controlled, AOA in the environment are exposed to fluctuating conditions in a complex ecosystem where substrates can often be limiting for growth (Table 2). There are knowledge gaps in how AOA respond to these environmental changes and which types of metabolisms are required for them to survive and thrive. Ammonia oxidation is highly specialised and not a widespread trait, and an argument could be made that there is little return on investing in alternative metabolisms. In contrast, ammonia oxidation offers little energetic reward and supplementing autotrophic growth could benefit nitrifiers, especially in oligotrophic environments, in environments where they co-exist with heterotrophs or in environments where ammonia oxidation is difficult, such as low oxygen conditions. For example, utilisation of small nitrogenous compounds such as urea and cyanate by AOA has been reported previously, but there are gaps in our understanding of the function and ecological importance of these pathways (Table 2). Likewise, it is known that compounds other than ammonia can interact with the archaeal AMO enzyme, but little is known about the potential role of alternative substrates and inhibitors of the AMO in the environment. Some of these substrates and inhibitors of the AMO occur naturally, but their influence on biogeochemical cycling is unknown (Table 2). An additional aspect of regulation and energy metabolism in ammonia oxidising microorganisms is that ammonia is both a source of energy as well as being required for constructing biomass. There must therefore presumably exist a system for sensing and regulating how ammonia is allocated within the cell (Table 2), particularly as ammonia oxidisers need to be able to rapidly respond to a changing environment, and to switch from growing to persisting or vice versa.

**Table 2:** Future perspectives.

**Ammonia uptake, assimilation, and oxidation**.
*Ammonia has a dual role in ammonia oxidisers, because it is required for both energy and building biomass*.
● How is the partitioning between ammonia assimilation and oxidation regulated, and does ammonium transport play a role in both?
**Co-oxidation of alternative substrates**.
*The archaeal ammonia monooxygenase (AMO) can interact with a range of compounds, including hydrocarbons*.
● Can the archaeal AMO also co-oxidise alternative substrates?
● What are the consequences of inhibition of, and co-oxidation by, AMO for biogeochemical cycling of nitrogen and other elements?
**Alternative energy yielding pathways**.
*Metabolic pathways, including energy conservation coupled to H*_*2*_ *production, have been predicted, but not yet validated*.
● Are these pathways functional, and under which environmental conditions do they operate?
*Some alternative types of metabolism, such utilisation of cyanate and urea, have been confirmed in AOA*.
● How important are the cyanate and urea metabolism in the adaptation of AOA to the environment?
**Linking metabolism and adaptation of AOA to their environments**.
*Environmental conditions, including ammonia and oxygen concentrations, fluctuate and ammonia oxidisers will need to adapt to these potentially sudden changes*.
● How do AOA sense and respond to these changes, and which metabolisms contribute to their success and resilience in the environment?
● Are AOA as metabolically inflexible as often thought?
● Do AOA use different strategies to grow and persist, and how important is dormancy in underpinning their cosmopolitanism?

The cultivation of ammonia oxidising microorganisms is notoriously difficult owing to their low yield and slow growth [139]. Nevertheless, validation of metabolism cannot be done from genetic repertoire alone. The study of isolated or highly enriched cultures is paramount to testing if the predicted metabolic pathways are functional, and to obtaining a mechanistic understanding of how they operate. The development of a genetic system for AOA would be especially valuable for investigating some of the putative metabolisms highlighted in this review and providing information about the regulation of major genes (Table 2). Furthermore, heterologous expression of AOA proteins could provide crucial insights on structure and biochemistry of key enzymes and transporters in vitro. In addition, single-cell and systems biology can deliver new knowledge which is not to easily accessible by other means. There is a promising outlook to further our understanding of nitrogen cycling and AOA by linking culture-based studies and culture-independent experiments on mixed communities from the environment. Novel mechanistic insights on the metabolism and biochemistry, including regulation, structure and function of key enzymes and discovery of new pathways, may help explain and predict how nitrifying communities respond to environmental changes, and how these factors together influence nitrification process rates in the environment.

## Supplementary information

Supplemental Material

## Data Availability

All data generated or analysed during this study are included in this published article and its supplementary information files.
