# Supplementary material for: Nitrification and beyond: metabolic versatility of ammonia oxidising archaea
Source: ISME J. 2023 Jul 14;17(9):1358–68. doi: 10.1038/s41396-023-01467-0 (PMC10432482; doi:10.1038/s41396-023-01467-0)
Supplement: Supplementary file 1 — Supplemental Material [file 41396_2023_1467_MOESM1_ESM.pdf]

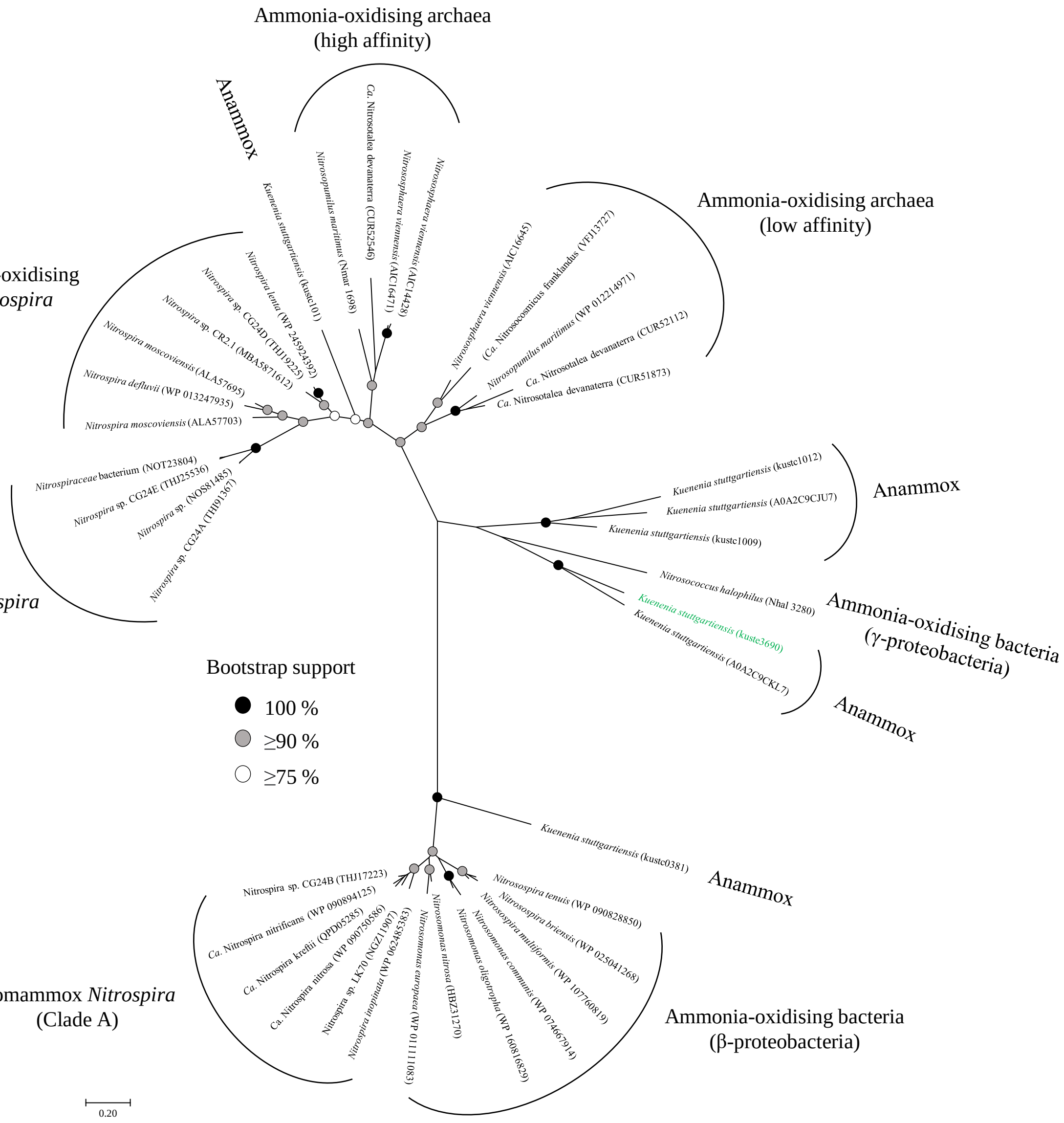

FIG S1 Maximum-likelihood phylogeny of Amt/Rh transporters encoded by ammonia oxidisers. Sequences were retrieved from NCBI and the phylogenetic tree constructed using maximum likelihood model of evolution with 100 bootstrap replicates (326 conserved amino acid residues) in MEGA software (1). All sequences in the tree are phylogenetically related, although they form distinct clusters. Green font in one of the sequences from *Kuenenia stuttgartiensis* refers to the ammonium sensor, which is phylogenetically related to Amt/Rh ammonium transporters but does not act as a transporter itself (2). The sequences in the upper part of the tree belong to Amt, the sequences in the bottom of the tree are Rh-type transporters.

References

1. Tamura K, Stecher G, Kumar S. MEGA11: molecular evolutionary genetics analysis version 11. Mol Biol Evol. 2021; 38:3022-7.

2. Pflüger T, Hernández CF, Lewe P, Frank F, Mertens H, Svergun D *et al*. Signaling ammonium across membranes through an ammonium sensor histidine kinase. Nat Commun. 2018; 9:164.
